# Supplementary material for: Disadvantages in preparing and publishing scientific papers caused by the dominance of the English language in science: The case of Colombian researchers in biological sciences
Source: PLoS One. 2020 Sep 16;15(9):e0238372. doi: 10.1371/journal.pone.0238372 (PMC7494110; doi:10.1371/journal.pone.0238372)
Supplement: S2 File — Questions in Spanish (original language) of the survey “Implications of language in scientific publications”. (PDF) [file pone.0238372.s002.pdf]

Implicaciones del idioma en las publicaciones científicas

Queridos(as) colegas,

Con esta encuesta están contribuyendo de manera anónima a una tesis de la Maestría en Comunicación Científica de la Universidad de Barcelona. El proyecto es sobre la importancia del multilingüismo en las publicaciones científicas, especialmente en el proceso de escritura de artículos.

Esta encuesta está dirigida a personas hispanohablantes (con enfásis en colombianos(as)) que estén realizando o hayan finalizado un doctorado en ciencias naturales y básicas (física, química, biología, biomedicina, etc).

Agradezco fuertemente su participación y su posterior difusión a otros(as) colegas.

*Obligatorio

Datos básicos

1. **Nacionalidad** *

2. **País en el que vive**
No ciudad o municipio

3. **Edad**

4. **Tipo de institución donde realizó sus estudios de bachillerato o secundaria**
Marca solo un óvalo.
- Colegio público
- Colegio privado

5. **Tipo de institución donde realizó sus estudios universitarios de grado**
Marca solo un óvalo.
- Universidad pública
- Universidad privada

6. **Escoja la ocupación a la que pertenece:**
Marca solo un óvalo por fila.

	Grandes empleadores, los altos directivos de las empresas y la Administración Pública, profesionales de alto nivel	Directivos y profesionales de nivel bajo	Empleados de nivel alto	Pequeños empleadores y por los trabajadores autónomos no profesionales	Trabajadores autónomos agrícolas	Supervisores y técnicos de rango inferior	Trabajadores del comercio y los servicios de rango inferior	Trabajadores manuales cualificados	Trabajadores no cualificados	Excluidos c mercado laboral, desemplead sin experienci previa
Madre										
Padre										
Abuelos(as) maternos										
Abuelos(as) paternos										
Hermano(a) 1										
Hermano(a) 2										
Hermano(a) 3										

7. **Estrato social de la vivienda (Solo si viven en Colombia)**
Marca solo un óvalo por fila.

	1	2	3	4	5	6
Usted						
Madre						
Padre						
Abuelos(as) paternos						
Abuelos(as) maternos						
Hermano(a) 1						
Hermano(a) 2						
Hermano(a) 3						

8. **Primera lengua(s)** *
En caso de ser bilingüe o trilingüe escriba 2 idiomas que principalmente usa

9. **Idioma(s) que domina**

10. **Nombre del programa o campo del doctorado que realiza o realizó** *

11. **Universidad o instituto dónde realiza o realizó el doctorado**

12. **Ya finalizó el doctorado?**
Marca solo un óvalo.
- Sí
- No

13. **Si ya finalizó el doctorado, cuál es su ocupación actual?**

14. **¿En qué año inició el doctorado?**

15. **Número aproximado de publicaciones académicas que tiene** *
Como autor principal y coautor. Artículos publicados, en revisión o aceptadas

16. **Porcentaje de sus publicaciones que ha realizado en INGLÉS** *
Como autor principal y coautor. Artículos publicados, en revisión o aceptadas
Marca solo un óvalo.
- Ninguna
- 10%
- 20%
- 30%
- 40%
- 50%
- 60%
- 70%
- 80%
- 90%
- 100%

17. **Porcentaje de sus publicaciones que ha publicado en su PRIMERA LENGUA**
Como autor principal y coautor. Artículos publicados, en revisión o aceptadas
Marca solo un óvalo.
- Ninguna
- 10%
- 20%
- 30%
- 40%
- 50%
- 60%
- 70%
- 80%
- 90%
- 100%

Escritura de artículos en inglés

18. **Una vez ya tiene completos los resultados de un estudio, cuánto tiempo se demora en el proceso de escritura del artículo si este se va a publicar en INGLÉS?**
Tiempo en días y número de horas diarias dedicadas. Ej: 20 días, 2 horas diarias

19. **Una vez ya tiene completos los resultados de un estudio, cuánto tiempo se demora en el proceso de escritura del artículo si este se va a publicar en su PRIMERA LENGUA?**
Tiempo en días y número de horas diarias dedicadas. Ej: 20 días, 2 horas diarias

20. **Al escribir un artículo que se va a publicar en inglés, usted preferentemente?** *
Marca solo un óvalo.
- Escribe en inglés directamente
- Escribe en su primera lengua y posteriormente hace la traducción al inglés

21. **Ha pedido favores o pagado por CORRECIÓN o REVISIÓN del inglés un artículo?** *
Sólo CORRECCIONES y EDICIÓN de texto, no traducciones
Marca solo un óvalo.
- Sí, he pedido favores
- Sí, he pagado
- Sí, ambas posibilidades
- No

22. **¿Cuál es el porcentaje de artículos por los cuales ha pedido favores o/y pagado por CORRECIÓN o REVISIÓN del inglés?**
Sólo CORRECCIONES y EDICIÓN de texto, no traducciones
Marca solo un óvalo por fila.

	Ninguno	10%	20%	30%	40%	50%	60%	70%	80%	100%
Pedido un favor										
Pagado										

23. **Si ha pagado, aproximadamente cuánto paga por cada artículo?**
Sólo CORRECCIONES y EDICIÓN de texto, no traducciones. En dólares

24. **Si ha pagado, cuál es la procedencia de ese dinero?**
Sólo CORRECCIONES y EDICIÓN de texto, no traducciones
Marca solo un óvalo.
- Únicamente yo
- Yo y los coautores
- Institución(es) académica(s)
- Beca(s) o subvención(es)
- Recursos mixtos

25. **Ha pedido favores o pagado por TRADUCIR un artículo al inglés?** *
Sólo traducciones
Marca solo un óvalo.
- Sí, he pedido favores
- Sí, he pagado
- Sí, ambas posibilidades
- No

26. **¿Cuál es el porcentaje de artículos por los cuales ha pedido favores o/y pagado por la TRADUCCIÓN al inglés?**
Sólo traducciones
Marca solo un óvalo por fila.

	Ninguno	10%	20%	30%	40%	50%	60%	70%	80%	100%
Pedido un favor										
Pagado										

27. **Si ha pagado, aproximadamente cuánto paga por cada artículo?**
Sólo TRADUCCIONES. En dólares

28. **Si ha pagado, cuál es la procedencia de ese dinero?**
Sólo TRADUCCIONES
Marca solo un óvalo.

- Únicamente yo
- Yo y los coautores
- Institución(es) académica(s)
- Beca(s) o subvención(es)
- Recursos mixtos

29. **Alguna vez ha dejado de participar (o de enviar el resumen para participar) en un congreso internacional por el hecho que debía hablar en inglés?**
Marca solo un óvalo.

- Sí
- No
- No quise una hacer presentación oral
- No quise presentar póster

30. **Califique que tan ansioso se siente por hablar en un congreso?**
Marca solo un óvalo por fila.

	Nada	Poco	Regular	Muy	Extremadamente
Presentando en inglés					
Presentado en su primera lengua					

31. **En el momento de leer un artículo científico en INGLÉS usted cree que ...**
Marca solo un óvalo por fila.

	Muy difícil	Difícil	Regular	Fácil	Muy fácil
Entender el texto en general					
Entender la terminología científica es					
Interpretar figuras y tablas es					

32. **En el momento de leer un artículo científico en PRIMERA LENGUA usted cree que ...**
Marca solo un óvalo por fila.

	Muy difícil	Difícil	Regular	Fácil	Muy fácil
Entender el texto en general					
Entender la terminología científica es					
Interpretar figuras y tablas es					

33. **En el momento de escribir un artículo científico en INGLÉS usted cree que ...**
Marca solo un óvalo por fila.

	Muy difícil	Difícil	Regular	Fácil	Muy fácil
Escribir la introducción es					
Escribir la metodología es					
Escribir los resultados es					
Escribir la discusión es					
Escribir la conclusión es					

34. **En el momento de escribir un artículo científico en su PRIMERA LENGUA usted cree que ...**
Marca solo un óvalo por fila.

	Muy difícil	Difícil	Regular	Fácil	Muy fácil
Escribir la introducción es					
Escribir la metodología es					
Escribir los resultados es					
Escribir la discusión es					
Escribir la conclusión es					

35. **Prefiere leer textos científicos en su primera lengua**
Marca solo un óvalo.

- Sí / Yes
- No

36. **Alguna revista le ha rechazado o solitado la correción de un artículo científico por una escritura incorrecta del inglés** *
Marca solo un óvalo.

- Sí / Yes
- No

37. **Sí tiene algún ejemplo de comentarios de los editores con respecto a la escritura incorrecta del inglés, por favor escríbalo**

Aprendizaje de inglés

38. **Qué porcentaje de inglés habla diariamente?**
Marca solo un óvalo.

- No hablo inglés diariamente
- 10%
- 20%
- 30%
- 40%
- 50%
- 60%
- 70%
- 80%
- 90%
- 100%

39. **Desde qué edad comenzó a aprender inglés?** *

40. **Cuántos años estudió o lleva estudiando inglés?** *

41. **Califique su experiencia de aprendizaje en la institución(es) donde estudió inglés** *
Marca solo un óvalo.

	1	2	3	4	5	
Mala						Muy buena

42. **Cuántos años ha vivido en un país angloparlante o que utilizará el inglés como primer idioma?**
Si nunca ha vivido coloque cero

43. **Califique su nivel de inglés actual** *
Marca solo un óvalo por fila.

	Excelente	Bueno	Regular	Malo
Escritura				
Habla				
Escucha				
Lectura				

44. **Ha presentado alguna examen de inglés con reconocimiento internacional?**
Marca solo un óvalo.

- Sí
- No

Con la tecnología de
Google Forms
