# Supplementary material for: Disadvantages in preparing and publishing scientific papers caused by the dominance of the English language in science: The case of Colombian researchers in biological sciences
Source: PLoS One. 2020 Sep 16;15(9):e0238372. doi: 10.1371/journal.pone.0238372 (PMC7494110; doi:10.1371/journal.pone.0238372)
Supplement: S5 File — Short explanation of English as lingua franca in Science, English as a foreign language in Colombia and Implication of English in Science (in English and Spanish). (PDF) [file pone.0238372.s005.pdf]

Theoretical Framework:

Disadvantages in preparing and publishing scientific papers caused by the dominance of the English language in science: The case of Colombian researchers in biological sciences

Valeria Ramírez-Castañeda

A short explanation of English as lingua franca in Science, English as a foreign language in Colombia, and the implication of English in Science (Traducción en español abajo).

English as lingua franca in science

Before 1900, the dominant languages in science were French, German, Italian, and Latin [1], currently 98% of scientific publications are in English. The global expansion of English has its origins in the second half of the 16th century with the reign of Queen Elizabeth [2]. Taking into account the so-called three circles of English by Braj Kachru's, before the XX century the first two circles were established [3]. The first characterized by the group of colonized countries where the massacre of its native population permit the establishment and expansion in the territory of European descendants mainly originating from Great Britain [3]. This circle includes countries where English is spoken as a first language (e.g. USA, Australia, New Zealand, England). The second circle contains the group of countries where colonialism was exercised mainly through the establishment of military and diplomatic campaigns, but where large groups of European settlers did not reside [3]. This second group includes countries where English is spoken as a second language and preserved as a vehicular language principally for commercial transactions (e.g.: India, Nigeria). The third circle includes all the countries where English is spoken as a foreign language. This expansion started at the end of the First World War when English began to be one of the most important languages in international politics. This is reflected in the

fact that the Versailles Treaty was versioned in English and French [4]. However, it was not until the end of the Second World War that English became global.

Until the second world war, only 50% of scientific publications were written in English [1]. However, at the end of the war, the fall of the European empires and the capitalist expansion consolidated the United States (US) as the only western power of the time [1]. Also, the stigmatization of the German language after the Second World War managed to secure English as the vehicular language for commercial, political and techno-scientific transactions in Europe [1]. This consolidation was promoted by the Marshall Plan in Europe and Japan, especially by the so-called cultural "Americanization" [5]. The US government saw in Europe a western ally in scientific, and technological innovation, this caused the US to re-open several German and French universities with American professors and scientists [1,5].

During the postwar period, science was used as a mechanism of mutual pressure between the two superpowers in formation, the Soviet Union and the USA. Consequently, Western science was published mainly in English and most of the scientific terms were coined in this language [6]. In 1969, 81% of publications in physics were written in English and this only continued to increase, when local magazines in countries such as Germany, Italy, and France, opened the doors to publication in English [1]. Beginning of the 1980s, English had already established itself as a *lingua franca* in the first world, a fact that increased the percentage of monolingual publications and conferences. On the other hand, university programs in countries such as German and the Netherlands began to be offered only in English [6].

Once the Soviet Union fell, more countries joined the learning of English as a foreign language, thus consolidating itself definitively as the most widely spoken language in the world [6]. At present, hundreds of laboratories and scientific events are conducted in English despite of being located in countries like Germany, Sweden, France, Switzerland, and Israel [7].

English as a foreign language in Colombia

The Colombian population is currently ranked number 60 out of 88 among global English levels, and in the last 8 years, it has fluctuated between low and very low levels [8]. Less than 1% of Colombians can be classified as bilingual in English, only the emigrating Colombians and Colombians with very high socioeconomic status belong to this category [9]. Even though that English has been taught as a second language in public and private institutions since 1994 [10], only 3% of the Colombian population uses English daily. Daily use only occurs in regions with a large tourist influx, but currently, it is becoming more and more relevant in work and academic contexts [11,12].

Teaching English is not mandatory by law and there is no official language proficiency test [13]. It was until 2004 that the Ministry of National Education implemented the National Program of Bilingualism (PNB) [14]. According to Fandiño-Parra [15], the PNB was designed to increase the country’s competencies in the neoliberal economy and globalization, the improvement of educational quality was a secondary objective. Hence, the PNB mentions [14]: “Being competent in another language is essential in the globalized world, which requires being able to communicate better, open borders, understand other contexts, appropriate knowledge and circulate it, understand and make ourselves understood, enrich and play a role decisive in the development of the country. Being bilingual expands opportunities to be more competent and competitive. " This type of strategy has already been implemented in other countries with to introduce cultural, economic, political, and social elements of US society, and can be interpreted as a form of control and imperialism attracted by the political and military intentions of the US governments [5,15]. Currently, this type of US intervention has led part of the population to identify English with the ideas of progress, innovation, modernity, and prosperity, however, another part of the population for historical reasons also associates the language with imperialism, exploitation, and domination. This influences that a part of the population feels contrary to the fact of learning English as a mandatory second language [11].

Implications of English in science

Two broad consequences, in some contradictory senses, can be derived from English as the only vehicle for science, 1) the globalization of scientific results based on the use of a single, “neutral” and “universal” language, 2) the inequality generated between native English-speaking countries or those with a high proficiency of language and the rest of the countries.

Firstly, the positive vision of the hegemonic use of English in science is strengthened with the idea that monolingualism generates cohesion among the scientific community. This idea argues that a monolingual science is fundamental for the dissemination of ideas, and up to recently, this argument has been accepted pramatically and not as a segregation factor [16,17]. In some countries such as Sweden, the use of English in scientific texts is versatile and depends on the type of audience more than on other political or ideological factors, which shows that speaking in English does not generate a fundamental problem for native Swedes [18]. In this same line, Ferguson et al [19] with their study conclude that even scientists from countries with a medium or low level of English, find a lingua franca in science necessity. They evaluate positively that this language is English and rejects the idea that it is a factor of inequality in science [20].

However, there is also a critical view of the hegemonic use of English in science. Comparing countries, there is a positive correlation between the number of researchers and publications and a higher English proficiency [8], highlighting the global inequality in scientific production in relation to the English language. This not only affects scientist but also society. For example, undergrad students in non-English-speaking countries receive a large amount of information in English, however, according to Lee & Fradd [21], science education depends on cultural attitudes derived from the first language such as the capacity for wonder, curiosity, persistence, and openness of ideas, among others. Learning using terms in another language and with foreign examples causes science to lose those cultural faculties, that is, science is alien to its own experiences. They approach

science differentially, they find more difficult to make reflections, retain information, and, therefore, obtain a good educational quality [22,23].

On the other hand, decreasing the dissemination of scientific knowledge in local languages causes gaps that are important for political, logistical, and economic decision-making within the territories. Amano et al [24] found that in Spain 54% of the people in charge at different levels of environmental management see the language as a barrier to understand and create strategies about their ecosystems. The last stronghold of the scientific content in languages other than English is local journals. These publications are related to low-quality science since the most important researcher is reserved for international journals [16]. By rejecting local publishers as a source of knowledge, it generates the false idea that all available and relevant knowledge is written in English. Ignoring relevant research worldwide could be catastrophic. A clear example of this is the case of two articles published in Chinese in January 2004, they warned of the avian flu and the H3N2 virus. These researchers concluded: "We should pay urgent attention about a possible pandemic outbreak of these two subtypes of influenza” [25]. However, it was not until August of the same year that international authorities paid attention to the report after the virus had already killed 100 million chickens and 26 humans [25]. On the other hand, Amano et al [24] reveal in their research that 35% of the documents related to the conservation of diversity are not published in English, a fact that generates biases in the knowledge and conservation of global ecosystems.

Despite the importance of local knowledge, for global south scientists, a successful career is correlated with academic migration. Scientists who never left their country of origin have fewer opportunities to publish in English, and therefore less recognition [15,16]. Learning to write, read, and speak in English is more difficult for scientists who stay in peripheral countries than for migrants [23,26,27]. Another consequence of the pressure to publish in international journals is that it increases economic expenses among EFL researchers. These expenses are related to editorial fees, translation, or so-called “ brokers ” services [28]. Curry & Lillis [28] showed that the authors prefer to use these translation or external

correction because of a sense of insecurity when writing becoming dependent on the accompaniment of NES researcher or editorial services [29,30].

References

1. Gordin MD. Scientific Babel. Scientific Babel. 2015. doi:10.7208/chicago/9780226000329.001.0001
2. Schwarz B. The Expansion of England. London: Routledge; 2005. doi:10.4324/9780203993187
3. Svartvik J, Leech G. English : One Tongue, Many Voices. 2nd ed. London: Palgrave Macmillan; 2016.
4. Palmowski J. A Dictionary of Contemporary World History. 3rd ed. A Dictionary of Contemporary World History. Oxford: Oxford University Press; 2014. doi:10.1093/acref/9780199295678.001.0001
5. Kieser A. The Americanization of Academic Management Education in Germany. J Manag Inq. 2004;13: 90–97. doi:10.1177/1056492604265301
6. Vítores F. El inglés en Europa: Origen y consolidación de una lengua franca. 2009; 57–69.
7. Ammon U. The dominance of English as a language of science : effects on other languages and language communities. Mouton de Gruyter; 2001.
8. EF Education. EF EPI 2018 - EF English Proficiency Index - Europe. In: EF Education First [Internet]. 2018 [cited 9 Jan 2019]. Available: https://www.ef.com.es/epi/
9. Andrés S-J. Biling□uismo en Colombia. Centro de Estudios Económicos Regionales. Banco de la República; 2015. doi:10.1186/s13256-015-0733-2
10. De Mejía A-M. Teaching English to Young Learners in Colombia: Policy, Practice and Challenges. MEXTESOL Journal. 2009.
11. Velez-Rendon G. English in Colombia: a sociolinguistic profile. World Englishes. 2003;22: 185–198. doi:10.1111/1467-971X.00287
12. Fandiño-Parra YJ, Bermúdez-Jiménez JR, Lugo-Vásquez VE. Retos del Programa Nacional de Bilingüismo: Colombia Bilingüe / Desafios do Programa Nacional de Bilinguismo: Colômbia Bilíngue / The Challenges Facing the National Program for Bilingualism: Bilingual Colombia. Educ y Educ VO - 15. 2012;15: 363–381. doi:http://dx.doi.org/10.5294/edu.2012.15.3.2
13. Cronquist K, Fiszbein A. El aprendizaje del inglés en América Latina. 2017.
14. Programa Nacional de Bilingüismo. Carta abierta L. Guías. 2006;
15. Fandiño-Parra YJ, Bermúdez-Jiménez JR, Lugo-Vásquez VE. The Challenges Facing the National Program for Bilingualism. Bilingual Colombia. Educ y Educ. 2012;15: 363–381. doi:10.5294/edu.2012.15.3.2
16. Salager-Meyer F. Scientific publishing in developing countries: Challenges for the future. J English Acad Purp. 2008; doi:10.1016/j.jeap.2008.03.009
17. Muresan L, Pérez-Llantada C. English for research publication and dissemination in bi-/multiliterate environments: The case of Romanian academics. J English Acad

Purp. 2014;13: 53–64. doi:10.1016/j.jeap.2013.10.009
18. McGrath L. Parallel language use in academic and outreach publication: A case study of policy and practice. J English Acad Purp. 2014;13: 5–16. doi:10.1016/j.jeap.2013.10.008
19. Ferguson G, Pérez-Llantada C, Plo R. English as an international language of scientific publication: a study of attitudes. World Englishes. 2011;30: 41–59. doi:10.1111/j.1467-971X.2010.01656.x
20. Bocanegra-Valle A. “English is my default academic language”: Voices from LSP scholars publishing in a multilingual journal. J English Acad Purp. 2014; doi:10.1016/j.jeap.2013.10.010
21. Lee O, Fradd SH. Science for All, Including Students From Non-English-Language Backgrounds. Educ Res. 2007;27: 12–21. doi:10.3102/0013189x027004012
22. Gulbrandsen P, Schroeder T V, Milerad J, Nylenna M. Paper or screen, mother tongue or English--which is better? Tidsskr Nor Laegeforen. 2002;122: 1646–8.
23. de Vasconcelos Hage SR, Cendes F, Montenegro MA, Abramides D V, Guimarães CA, Guerreiro MM. Specific language impairment: linguistic and neurobiological aspects. Arq Neuropsiquiatr. 2006;64: 173–80. doi:/S0004-282X2006000200001
24. Amano T, González-Varo JP, Sutherland WJ. Languages Are Still a Major Barrier to Global Science. PLoS Biol. 2016;14: e2000933. doi:10.1371/journal.pbio.2000933
25. Cyranoski D. Bird flu data languish in Chinese journals. Nature. 2004;430: 955–955. doi:10.1038/430955a
26. Benfield J, Howard K. The language of science. Eur J Cardio-Thoracic Surg. 2000;18: 642–648. doi:10.1016/S1010-7940(00)00595-9
27. Ferguson G. The global spread of English, scientific communication and ESP: questions of equity, access and domain loss. Ibérica Rev la Asoc Eur Lenguas para Fines Específicos (AELFE), ISSN 1139-7241, Nº 13, 2007, págs 7-38. 1999;13: 7–38.
28. Curry MJ, Lillis T. Multilingual Scholars and the Imperative to Publish in English: Negotiating Interests, Demands, and Rewards. TESOL Q. 2004; doi:10.2307/3588284
29. Shashok K. Editing around the World AuthorAID in the Eastern Mediterranean: a communication bridge between mainstream and emerging research communities. European Science Editing. 2009.
30. Pérez-Llantada C, Plo R, Ferguson GR. “You don’t say what you know, only what you can”: The perceptions and practices of senior Spanish academics regarding research dissemination in English. English Specif Purp. 2011;30: 18–30. doi:10.1016/j.esp.2010.05.001

Marco teórico

Breve explicación del inglés como lengua franca en la ciencia, inglés como lengua extranjera en Colombia y consecuencias del inglés en la ciencia (English translation above)

Inglés como lengua franca en la ciencia

Antes de 1900 las lenguas dominantes en la ciencia eran el francés, el alemán, el italiano y el latín (Gordin, 2015), en el presente el 98% de las publicaciones científicas son en inglés. La expansión global del inglés tiene sus orígenes en la segunda mitad del siglo XVI con el reinado de la reina Elizabeth (Schwarz, 2005). Teniendo en cuenta los llamados tres círculos del inglés de Braj Kachru, antes del siglo XX ya se habían establecido los dos primeros círculos. El primero caracterizado por el grupo de países colonizados por el imperio británico donde la masacre de su población nativa dio paso al establecimiento y expansión en el territorio de descendientes europeos principalmente originarios de Gran Bretaña (Svartvik & Leech, 2016). Este primer círculo incluye a los países donde se habla inglés como primera lengua (ej: EE. UU, Australia, Nueva Zelanda, Inglaterra). El segundo círculo está conformado por el grupo de países donde el colonialismo se ejerció principalmente por el establecimiento de campañas militares y diplomáticas, pero donde no residían grandes grupos de pobladores europeos (Svartvik & Leech, 2016). A este segundo grupo pertenecen los países donde el inglés se habla como segunda lengua y se conserva como lengua vehicular principalmente para transacciones comerciales (eg: India, Nigeria). El tercer círculo de expansión del inglés incluye a todos los países donde el inglés es utilizado como lengua extrajera. Este se posesiona a finales de la primera guerra mundial y comienza a ser una de las lenguas más importantes en la política internacional, hecho que se ve reflejado en que el tratado de Versalles fue versionado en inglés y francés (Palmowski, 2014). Sin embargo, no es hasta finales de segunda guerra mundial que el inglés alcanza un carácter global.

Hasta la segunda guerra mundial únicamente el 50% de las publicaciones científicas se escribía en inglés (Gordin, 2015). Sin embargo, a finales de la guerra, la caída de los imperios Europeos y de la expansión capitalista de EE.UU durante la guerra, lo consolidaron como la única potencia occidental de la época (Gordin, 2015). La reorganización económica y política que posicionó a Estados Unidos y a la Unión Soviética como las grandes potencias del mundo, además de la estigmatización del alemán a partir de la derrota del nazismo después de la segunda guerra mundial, lograron asegurar entre los países aliados el inglés como lengua vehicular principalmente a nivel comercial, político y tecno-científico (Gordin, 2015). Esta consolidación fue promovida por el plan Marshall en Europa y Japón especialmente por la llamada “americanización” cultural (Kieser, 2004). En ese momento EE.UU vio en Europa un aliado occidental en la innovación científica y tecnológica, en contraposición de los países comunistas, esto hizo

que EE.UU reinaugurara varias universidades alemanas y francesas con profesores y científicos estadounidenses (Kieser, 2004; Gordin, 2015).

Durante la posguerra, la ciencia fue utilizada como mecanismo de presión de las dos superpotencias en formación, la URSS y EE. UU, de donde se va a nutrir la carrera armamentista. En consecuencia, la ciencia producida en occidente se publicaba principalmente en inglés y la mayoría de los términos científicos se acuñaban en este idioma. El francés y el alemán como las lenguas más usadas en disciplinas como la química, la física y la psicología perdieron fuerza (Vítores, 2009). En 1969 el 81% de las publicaciones en física se escribían en inglés y esto solo siguió aumentando, las revistas locales de países como Alemania, Italia y Francia, abrieron las puertas a la publicación en inglés (Gordin, 2015). A partir de los años 80, el inglés ya se había consolidado como lingua franca en el primer mundo, hecho que aumentó el porcentaje de publicaciones y congresos monolingües. Por otro lado, programas universitarios en países como Alemania y Holanda se comenzaron a ofrecer únicamente en inglés (Vítores, 2009).

Una vez caída la Unión Soviética, más países se unieron al aprendizaje del inglés como EFL, consolidándose así definitivamente como la lengua más hablada en el mundo (Vítores, 2009). En el presente, cientos de laboratorios y eventos científicos se realizan en inglés a pesar de estar ubicados en países como Alemania, Suecia, Francia, Suiza e Israel (Ammon, 2001).

Inglés como lengua extrajera en Colombia

La población colombiana se posesiona actualmente de número 60 de 88 entre los niveles de inglés global. No obstante, en los últimos 8 años ha fluctuado entre los niveles bajo y muy bajo (EF Education, 2018). Menos del 1% de los colombianos se puede catalogar como bilingüe con el inglés, perteneciendo a esta categórica únicamente emigrantes a países angloparlantes o colombianos de nivel socioeconómico muy alto (Sanchéz-Jabba, 2013). A pesar de que el inglés se ha enseñado como segunda lengua en las instituciones públicas y privadas desde 1994 (de Mejía, 2009), únicamente el 3% de la población colombiana utiliza el inglés diariamente. Un uso diario principalmente ocurre en regiones de gran afluencia turística, y solo en la actualidad está comenzando a ser relevante en contextos laborales y académicos (Velez-Rendón, 2003; Fandiño-Parra, 2012).

La enseñanza del inglés no es obligatoria por ley y no existe ningún examen oficial del dominio del idioma (Cronquist & Fiszbein, 2017). No fue sino hasta el año 2004, que el Ministerio de Educación Nacional (MEN) implementó el Programa Nacional de Bilingüismo (PNB). Según Fandiño-Parra (2012), el PNB fue diseñado para que el país

aumentará sus competencias en la economía neoliberal y de la globalización, siendo el mejoramiento de la calidad educativa un objetivo secundario. Según el PNB (2006): "Ser competente en otra lengua es esencial en el mundo globalizado, el cual exige poderse comunicar mejor, abrir fronteras, comprender otros contextos, apropiar saberes y hacerlos circular, entender y hacerse entender, enriquecerse y jugar un papel decisivo en el desarrollo del país. Ser bilingüe amplía las oportunidades para ser más competentes y competitivos". Este tipo de estrategia ya ha sido implementada en otros países con la intención de introducir elementos culturales, económicos, políticos y sociales de la sociedad estadounidense en los países del mundo, y puede ser interpretado como una forma de control e imperialismo atraídos por los tratados de libre comercio, y por las intenciones políticas y militares de los gobiernos EE. UU en Colombia (Noble, 2006; Fandiño-Parra, 2012). Actualmente, este tipo de intervenciones estadounidenses han logrado que parte de la población identifique al inglés con las ideas de progreso, innovación, modernidad y prosperidad, sin embargo, otra parte de la población por motivos históricos también asocia el idioma con el imperialismo, la explotación y la dominación. Esto influye en que una parte de la población se sienta contraría con el hecho de aprender el inglés como segunda lengua obligatoria (Vélez-Rendón, 2003).

Implicaciones del inglés en la ciencia

Dos grandes consecuencias, en algunos sentidos contradictorias, se pueden derivar del inglés como única lengua vehicular de la ciencia. 1) La globalización de los resultados científicos a partir al uso de una lengua única, "neutra" y "universal", 2) La desigualdad generada entre los países angloparlantes nativos o con un alto nivel del idioma y el resto de países.

En primer lugar, la visión positiva del uso hegemónico del inglés en la ciencia se fortalece con la idea de que el monolingüismo genera cohesión entre la comunidad científica y que es fundamental para la difusión de ideas, además, entre la comunidad científica, este monolingüismo hasta ahora ha sido aceptado de manera pragmática y no como un factor de segregación (Salager-Meyes, 2006; Muresan & Pérez-Llantada, 2014). En algunos países como Suecia, el uso del inglés en los contextos científicos es versátil y depende del tipo de audiencia más que de otros factores políticos o ideológicos, que demuestra que el cambio al inglés no genera un problema de fondo en los nativos suecos (McGrath, 2014). Es esta misma línea, Ferguson y colaboradores (2011) con en su estudio concluyen que inclusive los científicos provenientes de países con nivel de inglés medio o bajo, encuentran como una necesidad un lenguaje universal en la ciencia, ven de manera positiva que ese lenguaje sea el inglés y rechazan la idea de que se trate de un

factor de desigualdad en la ciencia. Además, otros científicos encuentran oportuno el uso del inglés para difundir su trabajo nivel mundial (Bocanegra-Valle, 2014).

No obstante, también hay una visión crítica del uso hegemónico del inglés en la ciencia. Esta segunda posición se puede ver reflejada claramente en la figura del banco mundial donde muestra la correlación positiva entre número de investigadores y publicaciones y un mayor nivel de inglés (EF Education, 2018). Este es un factor de desigualdad y segregación que abarca diferentes actores involucrados en la producción científica y en el aprovechamiento de la ciencia. Por ejemplo, los estudiantes universitarios de ciencias en países no angloparlantes reciben gran cantidad de información en inglés, sin embargo, según Lee & Fradd (2007), la educación en ciencias depende de actitudes culturales derivadas a la primera lengua como la capacidad de asombro, la curiosidad, la persistencia, la apertura de ideas y el respeto a la ciencia, entre otras. Aprender con términos de otro idioma y ejemplos externos hace que la ciencia pierda esas facultades culturales, es decir, la ciencia es ajena a sus propias vivencias. Esta división aumenta en los estudiantes con un nivel de inglés bajo o carente, estos se acercan de manera diferencial a la ciencia, se les dificulta realizar reflexiones, retener información y, por lo tanto, obtener una buena calidad educativa (Gulbrandsen et al., 2002; de Vasconcelos, 2006).

Por otra parte, el conocimiento científico al dejar de estar disponible en los lenguajes locales ocasiona vacíos que son importantes para la toma de decisiones políticas, logísticas, económicas, entre otros, dentro de los territorios. Amano y colaboradores (2016) señalan que en España el 54% de las personas encargadas en distintos niveles del manejo ambiental ven al idioma como una barrera para entender y crear estrategias sobre sus propios ecosistemas. El último reducto de contenido científico investigativo en lenguas diferentes al inglés son las revistas locales, pequeñas y/o de países periféricos, estas publicaciones están relacionadas con ciencia de baja calidad dado que los grandes trabajos investigativos se reservan a las revistas internacionales (Salager-Meyes, 2006). Esto causa que los lectores con barreras lingüísticas solo accedan a este tipo de conocimiento y que inclusive desconozcan las grandes investigaciones que se realizan sobre su propia región (Salager-Meyes, 2006).

Al rechazar las pequeñas editoriales como fuente de conocimiento, dentro de la misma academia científica se genera una falsa idea de que todo el conocimiento disponible y relevante está escrito en inglés. Esto ocasiona que se ignoren a nivel mundial investigaciones relevantes que son publicadas en otros idiomas. Un claro ejemplo es el caso de dos artículos publicados en chino en enero del 2004, estos advertían del virus de la gripa aviar y del virus H3N2 y concluían con la frase “Se debe prestar atención urgente a la manifestación pandémica de estos dos subtipos de influenza” (Cyranoski,

2004). Sin embargo, no fue hasta agosto del mismo año que las autoridades internacionales prestaron atención al reporte, después de que el virus ya había ocasionado la muerte de 100 millones de gallinas y 26 humanos (Cyranoski, 2004). Por otra parte, Amano y colaboradores (2016) revelan en su investigación que el 35% de los documentos relacionados con la conservación de la diversidad no están publicados en inglés, hecho que genera sesgos en el conocimiento y conservación de los ecosistemas mundiales.

A pesar de la importancia del conocimiento local, el éxito profesional de un científico se correlaciona en mayor medida con su “internacionalización”. Esta presión constante podría estar influyendo en la migración académica, ya que los científicos que nunca dejaron su país de origen tienen menos oportunidades de publicar en inglés, y por lo tanto, menores oportunidades en la investigación científica. Posteriormente, los “expatriados”, por estas mismas razones, tienen mayores oportunidades de obtener puestos en su país local (Salanger-Meyer, 2008; Fandiño-Parra et al., 2012). Aprender a escribir, leer y hablar en inglés es más difícil para los científicos que se quedan en los países periféricos que para los migrantes (Benfield & Howard, 2000; Ferguson, 1999; Vasconcelos, 2006). Otra consecuencia de la presión de publicar en revistas internacionales es que aumenta el gasto económico entre los científicos no angloparlantes nativos de países por los servicios editoriales, de traducción o los llamados “brokers”. Curry & Lillis (2004) muestran que los “brokers” influyen en las oportunidades para publicar en revistas en inglés, por lo tanto, los autores pagan por estos servicios ya que son satisfactorios en su objetivo final. Los autores prefieren recurrir a estos servicios de traducción o corrección externos porque forja una sensación de inseguridad a la hora de escribir su investigación, ya que, los autores no angloparlantes nativos dudan más del vocabulario, sienten que no pueden formular frases complejas y en consecuencia se vuelven dependientes de un acompañamiento de investigadores nativos angloparlantes o de servicios editoriales (Shashok, 2009, Pérez-Llantada et al., 2011).

Referencias (Ver en sección en inglés “References”)
